# Membrane and Membrane Bioreactors Applied to Health and Life Sciences

**DOI:** 10.3390/membranes12060598

**Published:** 2022-06-09

**Authors:** Simona Salerno, Enrico Drioli, Loredana De Bartolo

**Affiliations:** 1 Institute on Membrane Technology, National Research Council of Italy (CNR-ITM), Via P. Bucci, Cubo 17/C, 87036 Rende, Italy; e.drioli@itm.cnr.it (E.D.); ldebartolo@itm.cnr.it (L.D.B.); 2 College of Chemical Engineering, Nanjing Tech University, Xinmofan Road, Nanjing 210009, China

The interest in membranes and membrane bioreactors for health and life sciences is rapidly growing thanks to their wide applications in advanced therapies and biotechnologies. Therapeutic approaches include the integration of membranes and membrane bioreactors for tissue regeneration and repair, for drug testing and drug delivery, and for cell therapy. Innovative biotechnologies employ membranes and membrane bioreactors for the production and selective separation of biological and bioactive molecules, for the design of diagnostic systems, and for the development of new drugs and pharmaceutical compounds.

This Special Issue collects six original research articles by renowned experts, highlighting the current advances and new trends in membrane preparation, the membrane separation process, membrane bioreactors, and membrane applications in tissue engineering and biotechnologies. 

A new sequential fabrication route of wet spinning combined with UV curing has been presented by Yin et al. [1] for the development of hierarchically organised collagen membranes that are customised to the requirements of healthcare applications, e.g., chronic wounds, tendon rupture, and orthopaedic defects. Despite its inherent bio-functionalities being critical to the clinical outcome, type I collagen is less prone to offer fibre control with respect to synthetic polymers, given its complex hierarchical organisation spanning from the nano- up to the macro-scale. Tronci’s research group addressed this challenge by designing bespoke type I collagen precursors bearing either single or multiple photoactive residues. Photoactive precursors that were wet-spun into fibres displayed the typical dichroic features of collagen and regular fibre morphology. The incubation of the wet-spun fibres in physiological conditions induced a nanoscale alignment and self-assembly of collagen-like D-periodic fibrils, which were successfully fixed following UV-induced network formation. The type and molar content of the photoactive residues covalently coupled to the collagen backbone enabled collagen hydrogels to be produced with varying hydrolytic degradation profiles and compression properties, together with a retained triple helix configuration. A remarkable combination of macroscopic properties was consequently observed in the wet spun collagen fibres. This work revealed that the selected manufacturing route from the molecular up to the microscale promptly generated new hierarchically assembled type I collagen fibres whereby material properties and collagen organisation can be controlled on demand by conditioning with either light or their physiological environment.

The use of membranes is of key interest in the development of engineered tissues. Membranes provide adaptable biomimetic microenvironments that can emulate the essential characteristics of the physiological ones, including tissue-specific extracellular matrix interactions. Surface properties trigger specific cell responses dictating the cell fate in terms of cell growth, migration, proliferation, differentiation and functional activation. Salerno et al. [2] developed CHT membranes tailoring the operational parameters in order to obtain nano- and micro-structured flat membranes with specific surface properties. The developed membranes were characterized and assessed for epidermal construction by using human keratinocytes to generate an epidermal strata model. The overall results demonstrated that the membrane surface properties strongly affected the stratification and terminal differentiation of human keratinocytes. It is noteworthy that human keratinocytes adhered on nanoporous CHT membranes, developing the structure of the corneum epidermal top layer, characterized by low thickness and low cell proliferation. On the microporous CHT membrane, keratinocytes formed an epidermal basal lamina, with high proliferating cells that stratified and differentiated over time, migrating along the *z*-axis and forming a multi-layered epidermis. This strategy represents an attractive tissue engineering approach for the creation of specific human epidermal strata to test the effects and toxicity of drugs, cosmetics, and pollutants.

Design optimization of membrane bioreactors for tissue engineering applications and upscaling analyses requires an in-depth knowledge and understanding of the mass transport of nutrients to ensure uniform distribution and to solve critical limitation issues. Wang et al. [3] investigated the glucose transport limitations in electrospun polycaprolactone scaffolds by using experimental data and mathematical modelling. Electrospun polycaprolactone membranes with different pore morphology, porosity, and tortuosity, which were induced by changing the electrospinning parameters, were prepared and utilized to provide a fundamental understanding of the effects of pore morphology on nutrient transport behaviour in hollow fibre membrane bioreactors. The glucose transport profile with dimensionless variables within the bioreactor was determined using the Krogh cylinder mode. The role of various dimensionless numbers (e.g., Péclet and Damköhler numbers) and non-dimensional groups of variables (e.g., non-dimensional fibre radius) on the glucose concentration profiles is discussed in this paper, highlighting the importance of these parameters in the design of a large-scale bioreactor for producing 3D bone tissues. 

The global biopharmaceutical market for therapeutic proteins, vaccines, and gene vector manufacturing requires reliable and efficient separation processes to ensure the safety, potency, and efficacy of the therapeutics. Membrane ion-exchange chromatography promises several advantages over column chromatography, including low operating pressure and fast flow rates, ease of use, and elimination of cleaning and validation costs in single-use applications. To this end, Lemma et al. [4] developed and characterized ion-exchange nonwoven membranes for the chromatographic purification of biomolecules. UV-grafted polybutylene terepthalate were prepared and functionalized with sulfonate and secondary amine for cation and anion exchange, respectively. The physical properties of the ion exchange membranes were measured to determine their porosity and flow properties, as well as their charged ligand densities, and how they affect the anion and cation ion exchange static and dynamic binding capacities. The cationic membranes purified human monoclonal antibodies and single-chain variable antibody fragments from cell culture supernatants with high yield and purity. The anionic membranes were able to remove high levels of DNA from protein solutions with little loss of the products. The purity of the eluted samples exceeded 97%, with good log-removal values for both host-cell proteins and DNA. Due to their excellent physical properties, high binding capacity and selectivity, low operating pressure dropped at high linear velocities, and high productivity in a bind-and-elute mode, this type of membrane showed significant promise for single-use purification devices in biopharmaceutical manufacturing.

Membrane emulsification is a promising methodology in cosmetic and pharmaceutical biotechnology, with unique properties in terms of precise manufacturing of emulsion droplets under mild operating conditions, suitable to preserve the stability of bioactive labile components. Piacentini et al. [5] used membrane emulsification technology for the production of a microstructured emulsion bioreactor using lipase as a catalyst and as a surfactant at the same time. Compared to the classical methodology used to separate enantiomers (i.e., diastereomeric crystallization), the kinetic resolution in multiphase reaction systems implemented by membrane emulsification proved to have great efficiency for optically pure enantiomer production. The enzyme loaded at the stable emulsion interface showed very high enantioselectivity (100%) and unprecedented (S)-substrate conversion (100%). The controlled formulation of uniform and stable droplets permitted the evaluation of the lipase amount distributed at the interface, and therefore the evaluation of enzyme-specific activity as well as the estimation of the hydrodynamic radius of the enzyme at the oil/water (o/w) interface in its maximum enantioselectivity. This paper demonstrated that the membrane emulsification method also allows for the evaluation of the size of biocatalysts with interfacial activity in their catalytic enantioselective conformation, which is a key point in bioreactor construction. 

Novel approaches in membrane technology aim to overcome the main drawback of filtration and separation processes, i.e., membrane fouling. This phenomenon, caused by the accumulation of solutes on the membrane surface or within the membrane pore structure, irreversibly affects the performance of the membranes and increases the overall operational cost due to the need for regular membrane-cleaning procedures. Among the different antifouling strategies that have been employed, magnetic responsive membranes allow for the control of solute-membrane interactions through a reversible adjustment and modification of physicochemical properties of the membrane surface, which translate into tuneable permeate fluxes and superior antifouling characteristics. Upadhyaya et al. [6] developed block copolymer-based magnetic mixed matrix nanocomposite membranes and assessed the effect and impact of a magnetic field in mitigating biofouling in bioseparation processes. The hydrophilic nanocomposite membranes were composed of spherical polymeric nanoparticles (NPs) synthesized through polymerization-induced self-assembly (PISA) with iron oxide. The hydrophobic nanocomposite membranes were prepared via nonsolvent-induced phase separation (NIPS) containing poly (methacrylic acid) and meso-2,3-dimercaptosuccinic acid-coated superparamagnetic nanoparticles (SPNPs). Such membranes showed promising results for better control over membrane biofouling using a non-energy-intensive magnetic field. Indeed, the suitable manipulation of an external magnetic field—by a cyclic variation of the field intensity—resulted in structural rearrangements taking place in the top layer of the membrane, which were reversible to a certain extent, and which modulated membrane transport and protein transmission with an impact on the permeate fluxes and the protein-sieving coefficients. 

In conclusion, the different contributions collected in this Special Issue highlight the multifunctional role of membranes and membrane bioreactors in health and life science applications, addressing critical issues and aspects of the field. 

As guest editors, we would like to thank all the authors for their outstanding contributions. We sincerely feel that overall these papers offer innovative approaches and new trends that could stimulate future research in membrane science applied to the biomedical and pharmaceutical field. We would also like to thank the reviewers for their valuable contributions and the editorial staff of *Membranes* for their help and support during the review process.
